# Ethane Chlorination Toward Vinyl Chloride Synthesis: Mechanistic and Catalytic Perspectives

**DOI:** 10.1002/anie.202523506

**Published:** 2026-02-02

**Authors:** Xia Wu, Guodong Huo, Haifeng Qi, Qinggang Liu, Nicholas F. Dummer, Graham J. Hutchings, Yanqiang Huang

**Affiliations:** ^1^ State Key Laboratory of Catalysis Dalian Institute of Chemical Physics Chinese Academy of Sciences Dalian China; ^2^ Chemical Engineering and Resource Utilization Northeast Forestry University Harbin China; ^3^ Max Planck‐Cardiff Centre on the Fundamentals of Heterogeneous Catalysis FUNCAT Cardiff Catalysis Institute Translational Research Hub Cardiff University Cardiff UK

**Keywords:** ethane chlorination, heterogeneous catalysis, radical chemistry, rare‐earth oxychlorides, vinyl chloride monomer

## Abstract

Ethane chlorination has emerged as a promising alternative to conventional ethylene‐ and acetylene‐based routes for the production of vinyl chloride monomer (VCM). Unlike conventional catalytic processes, this approach relies on chlorine radical‐mediated activation to convert ethane into 1,2‐dichloroethane, followed by thermal cracking to VCM. However, this route remains in its early stages, hindered by the complexity of gas‐phase radical chemistry and catalyst deactivation under chlorination conditions. This review provides a critical assessment of the mechanistic foundations of ethane chlorination, highlighting the interplay between radical‐mediated and surface‐catalyzed pathways. Particular attention is given to advances in rare‐earth oxychloride catalysts, which have shown the ability to stabilize key intermediates. We also discuss major deactivation mechanisms, including phase transformation and surface hydroxylation, that limit catalyst lifetime. Furthermore, we highlight the feasibility of ethane chlorination as a low‐carbon VCM production route under future decarbonized energy scenarios. Finally, key directions in catalyst design, mechanistic understanding, and process integration are outlined to advance ethane chlorination from laboratory‐scale innovation to industrial reality.

## Graphical abstract

Ethane chlorination is emerging as a promising low‐carbon route to vinyl chloride monomer, yet it remains academically underexplored due to the complexity of radical chemistry. As the first focused Minireview on this topic, it offers forward‐looking mechanistic analysis, unveils catalyst evolution pathways, and defines research priorities to accelerate breakthroughs in this nascent but critical field.

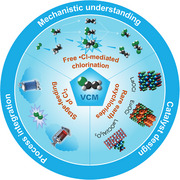

## 1 Introduction

**FIGURE 1:**
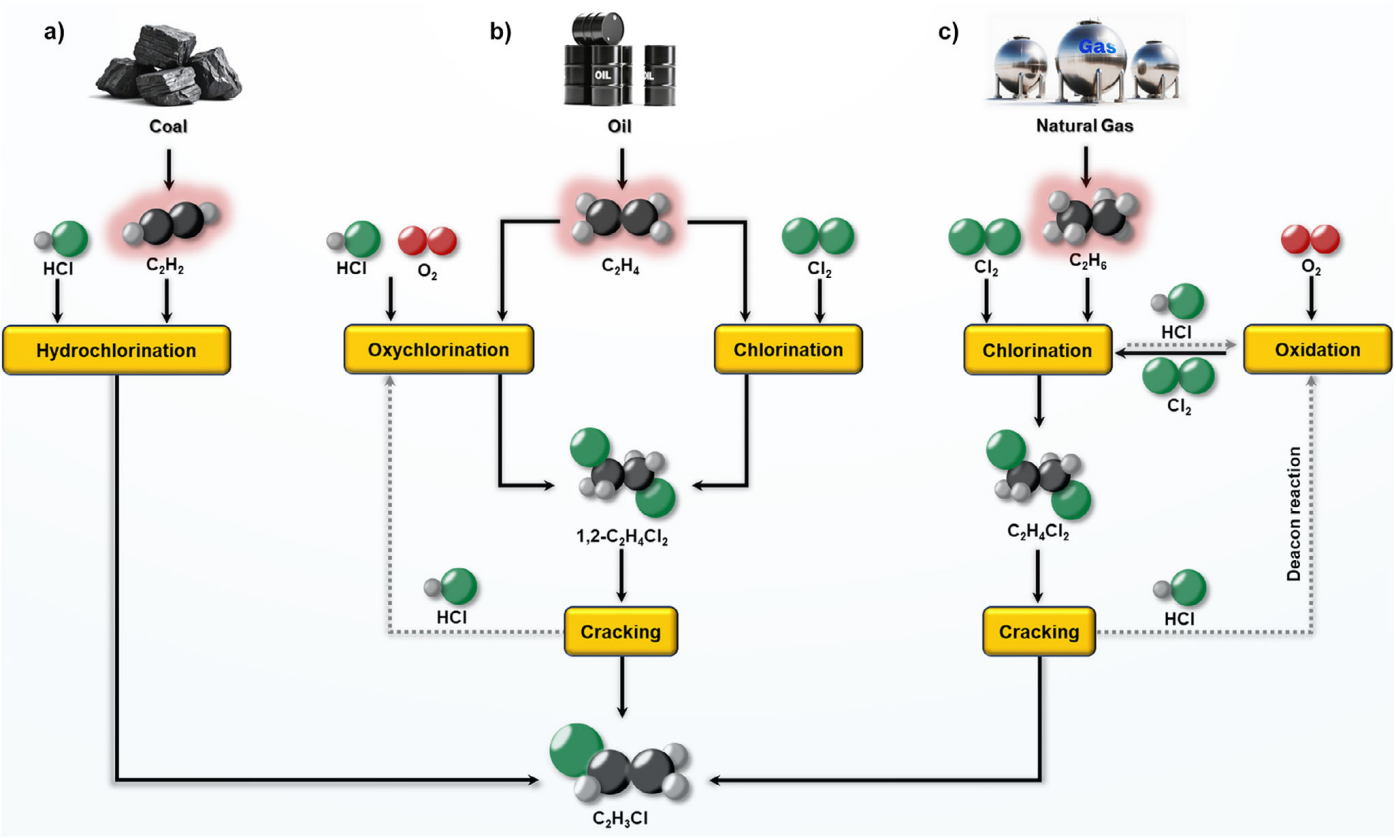
Schematic illustration of VCM synthesis via C_2_H_2_ hydrochlorination (a), the C_2_H_4_‐based balanced process (b), and C_2_H_6_ chlorination (c).

Polyvinyl chloride (PVC), the third most produced synthetic polymer, plays a crucial role in applications ranging from construction materials to medical devices [1, 2, 3, 4, 5]. Its precursor, vinyl chloride monomer (VCM), is currently synthesized through the acetylene (C_2_H_2_) hydrochlorination and the ethylene (C_2_H_4_) balanced process (Figure 1a,b, respectively) [6, 7, 8, 9, 10, 11]. While the former still relies on mercury‐containing catalysts with severe environmental and health consequences [12, 13], the latter depends on oil‐derived C_2_H_4_ and entails a multistep process involving chlorination, oxychlorination, and thermal cracking [14, 15, 16, 17, 18, 19, 20, 21, 22]. Both traditional routes are energy‐ and carbon‐intensive, with the C_2_H_4_‐based pathway emitting ∼2 kg CO_2_‐eq kg^−1^ of VCM [23]. Coupled with rising oil prices and tightening regulations on carbon and mercury emissions, these processes have become increasingly unsustainable [24, 25].

Accordingly, increasing attention has turned to the direct chlorination of light alkanes as an alternative strategy for VCM production. Building on insights gained from methane (CH_4_) chlorination, where selective C–Cl bond formation from Cl_2_ has been demonstrated under catalytic conditions [26, 27], ethane (C_2_H_6_) has emerged as a particularly attractive feedstock [28, 29, 30, 31, 32, 33]. Chlorination of C_2_H_6_ to 1,2‐dichloroethane (1,2‐C_2_H_4_Cl_2_), followed by its established thermal cracking to VCM, offers a promising route that bypasses the need for C_2_H_4_ or C_2_H_2_ (Figure 1c) [34]. Notably, as the cracking step is already a commercially proven and well‐optimized technology, research efforts can focus primarily on improving the selective chlorination of C_2_H_6_ rather than reinventing the entire VCM production chain. Compared to C_2_H_4_‐based routes, C_2_H_6_ chlorination has been projected to reduce production costs by over 30% under current conditions [23].

Despite its advantages, the industrial implementation of C_2_H_6_ chlorination remains challenging due to the complex nature of its reaction chemistry. The conversion of C_2_H_6_ to 1,2‐C_2_H_4_Cl_2_ is predominantly governed by radical mechanisms initiated by chlorine radicals (•Cl). Hydrogen atoms are abstracted from C_2_H_6_ by •Cl, generating ethyl radicals that further react with •Cl to form chloroethane (C_2_H_5_Cl) and ultimately dichlorinated products [35]. While efficient at initiating C–H activation, •Cl radicals are highly unselective, often promoting over‐chlorination and dehydrochlorination side reactions. As a result, maintaining product selectivity becomes increasingly difficult at higher C_2_H_6_ conversions.

Heterogeneous catalysts—particularly rare‐earth oxychlorides like LaOCl and EuOCl—have been explored to modulate radical reactivity and improve selectivity [36, 37]. These catalysts offer sites for •Cl stabilization and facilitate regioselective hydrogen abstraction. However, under Cl_2_‐rich conditions, LaOCl is prone to irreversible transformation into LaCl_3_, a phase with significantly diminished catalytic activity. Additional challenges such as surface hydroxylation and sintering further degrade catalyst performance and shift product distribution toward undesired species [35]. Adding to the complexity is the multifaceted nature of the reaction system, where gas‐phase radical chemistry and surface‐mediated catalysis coexist and interact dynamically. This duality makes it difficult to decouple kinetic effects, complicates reactor design, and limits the effectiveness of conventional catalytic control strategies. Achieving industrially relevant performance will require a new generation of materials informed by a fundamental understanding of radical–surface interactions.

In light of these considerations, this review aims to provide a comprehensive and critical assessment of C_2_H_6_ chlorination as a platform for low‐carbon VCM production. We begin by outlining the mechanistic foundations of gas‐phase •Cl‐mediated C_2_H_6_ chlorination, followed by a discussion of recent advances in catalyst design, structure–function relationships, and deactivation mechanisms. We then evaluate the techno‐economic and environmental performance of C_2_H_6_‐based routes in comparison to conventional technologies. Finally, we highlight key challenges and research priorities that need to be addressed to enable the industrial realization of this emerging process. By providing a unified view of reaction mechanisms and catalyst design principles, this work offers a comprehensive foundation for the development of next‐generation chlorination technologies centered on natural gas feedstocks.

## 2 Ethane Chlorination Chemistry

### 2.1 Reaction Thermodynamics of C_2_H_6_ Chlorination

The chlorination of C_2_H_6_ to 1,2‐C_2_H_4_Cl_2_ involves two consecutive radical‐mediated steps, with hydrogen chloride (HCl) as a co‐product. The balanced overall reactions are:

$$C_{2}H_{6}+{\text{Cl}}_{2}\to C_{2}H_{5}\text{Cl}+\text{HCl},\Delta H_{303K}^{o}=-142\;\text{kJ}\;{\text{mol}}^{-1}$$

$$C_{2}H_{5}\text{Cl}+{\text{Cl}}_{2}\to 1,2-C_{2}H_{4}{\text{Cl}}_{2}+\text{HCl},\Delta H_{303K}^{o}=-131\;\text{kJ}\;{\text{mol}}^{-1}$$

Alternatively, the net reaction can be summarized as:

$$C_{2}H_{6}+2{\text{Cl}}_{2}\to 1,2-C_{2}H_{4}{\text{Cl}}_{2}+2\text{HCl},\Delta H_{303K}^{o}=-273\;\text{kJ}\;{\text{mol}}^{-1}$$

In conventional C_2_H_4_‐based VCM production, HCl is typically recycled via oxychlorination:

$$C_{2}H_{4}+2\text{HCl}+\frac{1}{2}O_{2}\to C_{2}H_{4}{\text{Cl}}_{2}+H_{2}O,\Delta H_{303K}^{o}=-566\;\text{kJ}\;{\text{mol}}^{-1}$$

For C_2_H_6_ chlorination, a similar HCl recovery loop can be implemented, as discussed in Section 4:

$$2\text{HCl}+\frac{1}{2}O_{2}\to {\text{Cl}}_{2}+H_{2}O,\Delta H_{303K}^{o}=-114\;\text{kJ}\;{\text{mol}}^{-1}$$

These thermodynamic data clearly indicate that C_2_H_6_ chlorination is strongly exothermic, releasing substantial heat that must be carefully managed in reactor design to ensure safe and efficient operation.

### 2.2 Mechanistic Foundations of C_2_H_6_ Chlorination

The chlorination of C_2_H_6_ is predominantly governed by a radical chain mechanism. The initiation of •Cl can proceed via two principal pathways depending on the reaction conditions. Under non‐catalytic conditions, •Cl is generated through thermal homolysis of Cl_2_. This step has a high activation energy and thus becomes significant only at elevated temperatures (typically > 200 °C). In contrast, in the presence of a heterogeneous catalyst, a lower‐energy pathway emerges. Catalytic surfaces, particularly those of rare‐earth oxychlorides, can facilitate catalyst‐assisted Cl_2_ activation at moderate temperatures. This involves the heterolytic or homolytic cleavage of the Cl–Cl bond at surface sites, leading to the formation of adsorbed chlorine species that can readily desorb or participate in surface reactions.

**FIGURE 2:**
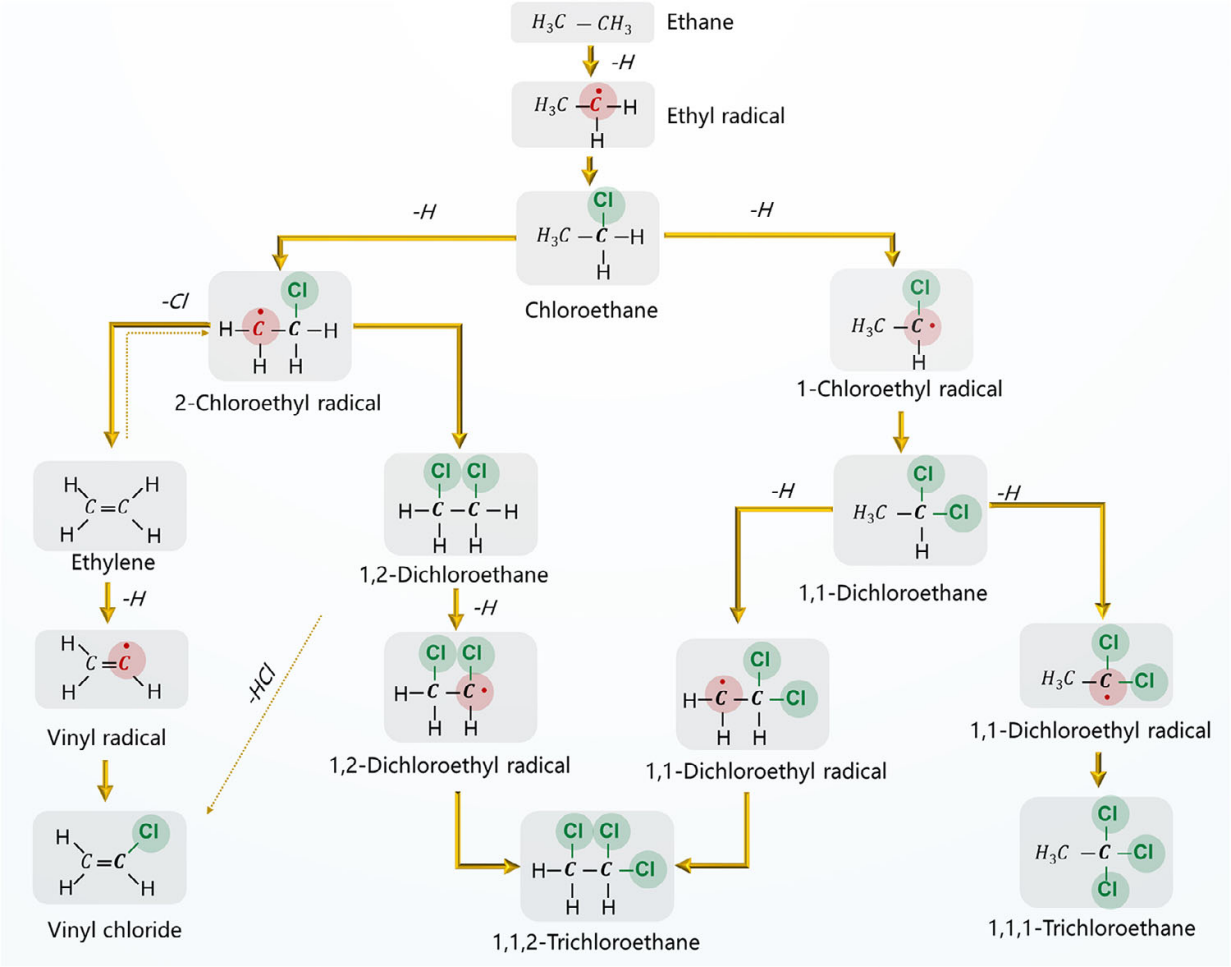
Proposed reaction pathways for C_2_H_6_ chlorination via chlorine radical mechanisms.

Once generated, •Cl radicals rapidly abstract a hydrogen atom from C_2_H_6_ to form an ethyl radical (•C_2_H_5_) and HCl [38]. These •C_2_H_5_ then react with •Cl to produce C_2_H_5_Cl. Upon further chlorination, C_2_H_5_Cl is converted into dichloroethane through similar radical steps. The distribution of isomers, 1,1‐C_2_H_4_Cl_2_ and 1,2‐C_2_H_4_Cl_2_, is dictated by both kinetic and thermodynamic considerations. Specifically, the 1‐chloroethyl radical (leading to 1,1‐C_2_H_4_Cl_2_) is more stable by approximately 15 kJ mol^−1^ than the 2‐chloroethyl radical (leading to 1,2‐C_2_H_4_Cl_2_), resulting in a strong preference for 1,1‐isomer formation under non‐catalytic conditions [35]. This reaction network is depicted in Figure 2, which outlines the radical‐mediated conversion of C_2_H_6_ to mono‐ and multi‐chlorinated products via hydrogen abstraction and chlorine substitution.

**FIGURE 3:**
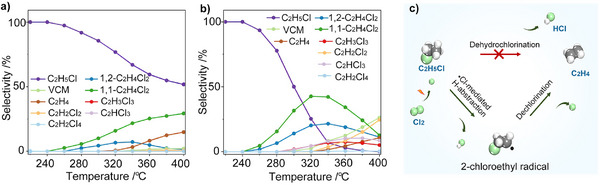
Temperature‐induced changes in radical reaction pathways during C_2_H_6_ chlorination under different feed compositions: (a) C_2_H_6_/Cl_2_/N_2_ = 4:4:92 (vol.%); (b) C_2_H_6_/Cl_2_/N_2_ = 4:8:88 (vol.%). (c) Proposed mechanism for •Cl‐mediated dehydrochlorination of C_2_H_5_Cl to C_2_H_4_ via 2‐chloroethyl radical intermediates. Reprinted from ref [39] with permission.

Thermodynamic and kinetic analyses further support the dominance of •Cl‐driven steps. Quantum chemical calculations reveal that hydrogen abstraction from C_2_H_6_ by •Cl has a low activation energy of 2.1 kJ mol^−1^ and is mildly exothermic (Δ*H* = –3.8 kJ mol^−1^) [38]. In the case of C_2_H_5_Cl, hydrogen abstraction occurs without an energy barrier to form •CHClCH_3_ (*E*_a_ = 0 kJ mol^−1^), whereas the alternative pathway leading to •CH_2_CH_2_Cl is also accessible but requires a higher activation energy (*E*_a_ = 8.9 kJ mol^−1^) [38]. These values explain why dichlorination becomes increasingly selective toward the 1,1‐isomer under thermal conditions. Experimental observations agree with this theoretical prediction. In the absence of a catalyst, the product distribution of dichloroethane isomers consistently favors 1,1‐C_2_H_4_Cl_2_, with a typical molar ratio of 2:1 relative to the 1,2‐isomer, invariant across a wide temperature range (250 °C–320 °C) and under various Cl_2_ concentrations (Figure 3a,b) [39].

Interestingly, at temperatures exceeding 320 °C, new kinetic pathways become competitive under Cl_2_‐lean conditions (C_2_H_6_/Cl_2_ molar ratio of 4:4). One such pathway involves the decomposition of the 2‐chloroethyl radical, formed during the chlorination of C_2_H_5_Cl, to C_2_H_4_ and a •Cl, rather than undergoing further chlorination to produce 1,2‐C_2_H_4_Cl_2_ (Figure 3a) [39]. This pathway leads to the formation of C_2_H_4_, which becomes a dominant byproduct when the Cl_2_ concentration is insufficient to sustain sequential chlorination. Thermal decomposition studies demonstrate that C_2_H_5_Cl remains stable even at 400 °C in the absence of Cl_2_, confirming that C_2_H_4_ generation is not a consequence of direct thermal cleavage. Instead, C_2_H_4_ formation is mediated by chlorine radicals, which facilitate *α*‐C–Cl bond scission through hydrogen abstraction (Figure 3c). This mechanistic bifurcation highlights the dual role of •Cl species, serving both as chlorinating agents and as drivers of dehydrochlorination, depending critically on their local concentration.

Moreover, the chlorination reactivity of C_2_H_6_ and its intermediates shows a clear declining trend with increasing chlorine substitution. The reactivity sequence under identical thermal conditions follows: C_2_H_6_ > C_2_H_5_Cl > C_2_H_4_Cl_2_ > C_2_H_3_Cl_3_. The primary factor responsible is a progressive decrease in the pre‐exponential factor (*A*) in the Arrhenius expression [38]. This decline stems from increased molecular mass and moment of inertia upon chlorination, which lowers the effective frequency of reactive collisions. The chlorination of 1,2‐C_2_H_4_Cl_2_ is particularly slow, which reinforces the experimental observation that over‐chlorination generally originates from 1,1‐isomers rather than 1,2‐isomers.

Despite the inherent exothermicity and favorable kinetics of C_2_H_6_ chlorination, the process lacks sufficient selectivity in the absence of catalytic control. The •Cl radical is highly reactive and non‐discriminatory, such that over‐chlorination and chain branching cannot be suppressed thermally. Therefore, while gas‐phase radical chemistry provides an efficient activation strategy for inert C–H bonds, it also introduces selectivity challenges and complicates process scalability.

## 3 Oxychloride‐Based Catalysts for Selective C_2_H_6_ Chlorination

### 3.1 Catalyst Design and Structural Evolution

**FIGURE 4:**
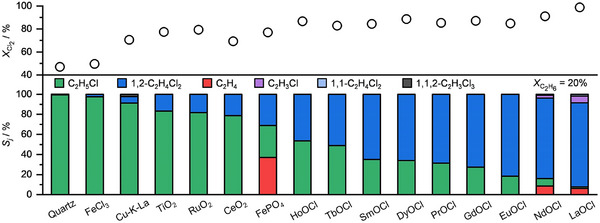
Cl_2_ conversion (top) and selectivity to product *j* (bottom) in C_2_H_6_ chlorination over the catalysts at approximately 20% C_2_H_6_ conversion. Reaction conditions: C_2_H_6_:Cl_2_:Ar:He = 6:3:4.5:86.5, 287 °C–300 °C, 1 bar. Reprinted from ref [40]; Copyright 2021, John Wiley and Sons.

Catalyst design for C_2_H_6_ chlorination plays a pivotal role not only in shifting product selectivity from undesired over‐chlorinated species to target 1,2‐C_2_H_4_Cl_2_, but also in enabling long‐term structural stability under aggressive Cl_2_‐rich conditions. Rare‐earth oxychlorides, particularly LaOCl and EuOCl, have emerged as highly promising catalytic materials due to their ability to mediate Cl_2_ activation and facilitate the transformation of intermediate species such as C_2_H_5_Cl into 1,2‐C_2_H_4_Cl_2_ (Figure 4) [40]. These catalysts exhibit high selectivity (> 80%) and sustained stability over extended operation periods under Cl_2_‐lean conditions (C_2_H_6_/Cl_2_ molar ratio of 6:3).

**FIGURE 5:**
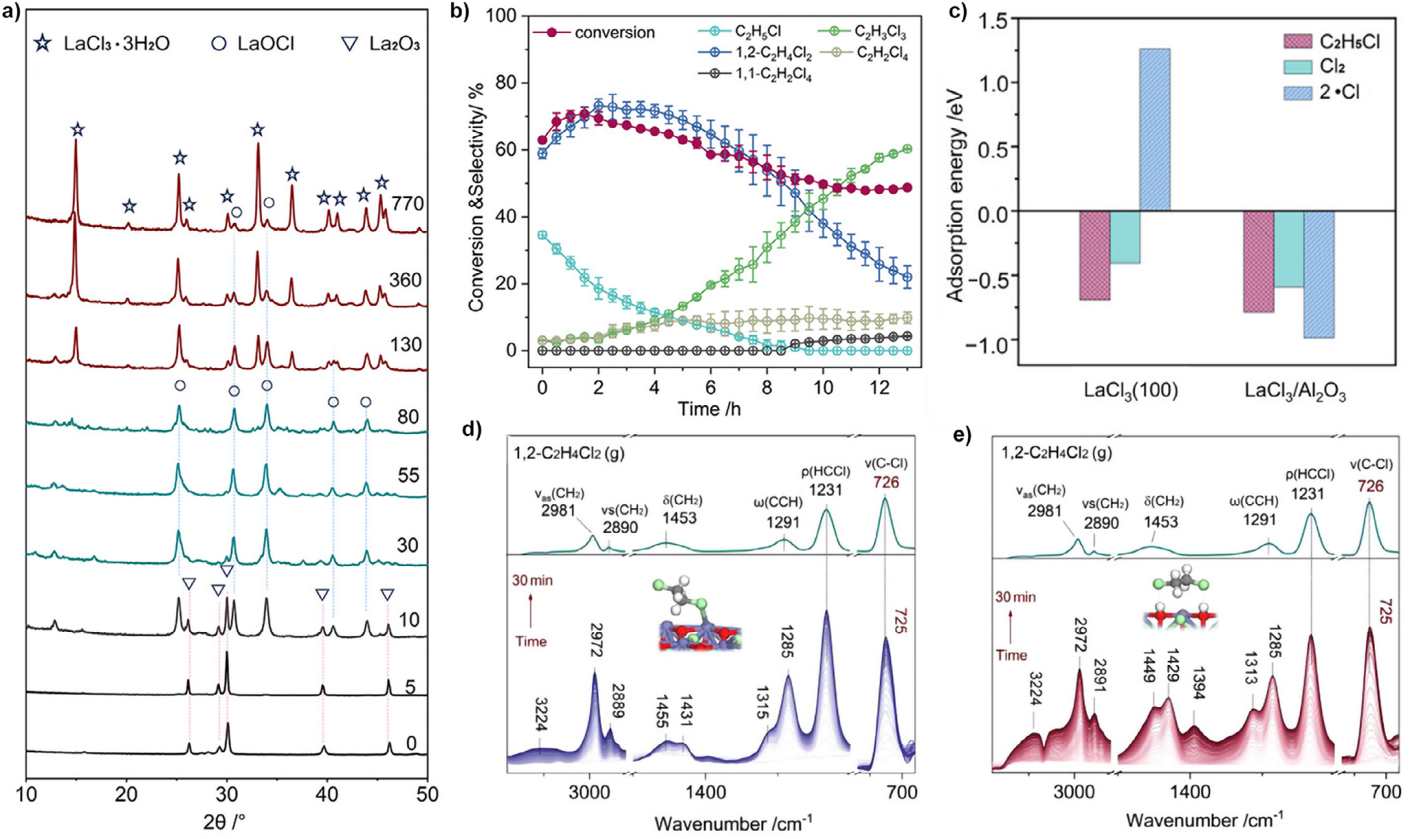
(a) Time‐resolved XRD patterns showing structural evolution of the La_2_O_3_ catalyst under reaction atmosphere. (b) Conversion and selectivity of C_2_H_6_ chlorination over the La_2_O_3_ catalyst. Reaction conditions: C_2_H_6_/Cl_2_/N_2_ = 4:9:87, 260 °C. (c) Adsorption energies of surface species on the sites of LaCl_3_/Al_2_O_3_ and LaCl_3_(100). In situ DRIFTS of 1,2‐C_2_H_4_Cl_2_ adsorption on La catalysts sampled at 55 min (d) and 770 min (e). Reprinted from ref [37] with permission.

Despite these advantages, structural evolution poses a significant challenge to the long‐term selectivity of bulk oxychloride catalysts under industrially relevant conditions. Phase evolution studies have shown that under Cl_2_‐rich conditions (C_2_H_6_/Cl_2_ molar ratio of 4:9), materials such as LaOCl progressively transform into LaCl_3_ (LaOCl + 2HCl ⟶ LaCl_3_ + H_2_O, Figure 5a) [37]. As LaOCl constitutes the primary active phase responsible for the selective formation of 1,2‐C_2_H_4_Cl_2_, its structural degradation to LaCl_3_ is strongly associated with a decline in 1,2‐C_2_H_4_Cl_2_ selectivity and a concomitant increase in over‐chlorinated byproducts (Figure 5b). Interestingly, contrary to the postulate that the Lewis acidity of LaCl_3_ might promote over‐chlorination, a model catalyst study (LaCl_3_/Al_2_O_3_) and theoretical calculations (Figure 5c) demonstrate that aggregated LaCl_3_ nanoparticles lack the ability to stabilize •Cl and consequently deactivate the surface sites, indicating that LaCl_3_ formation itself is not the driving force behind 1,2‐C_2_H_4_Cl_2_ over‐chlorination. Instead, it is primarily attributable to the accumulation of surface hydroxyl groups (–OH) during the phase transformation process. Specifically, surface –OH species enable bidentate adsorption of 1,2‐C_2_H_4_Cl_2_ through hydrogen bonding (Figure 5d,e), significantly increasing its adsorption strength (Δ*E*_ad_ = –1.35 eV, compared to –0.65 eV on hydroxyl‐free surfaces). This enhanced adsorption facilitates the activation of C–Cl bonds and overcomes the energy barrier for subsequent chlorination. Therefore, the structural evolution of rare‐earth oxychlorides under Cl_2_‐rich conditions, accompanied by hydroxyl accumulation, represents a critical bottleneck in maintaining long‐term selectivity.

To address these limitations, recent studies have explored confinement strategies, with notable success achieved using Al_2_O_3_ as a structural stabilizer [41]. When La is introduced onto γ‐Al_2_O_3_ via co‐precipitation followed by calcination at 900 °C, the resulting LaAl_2_O_3_‐like composite exhibits highly dispersed La species and strong La–O–Al interfacial bonding. The formation of La–O–Al linkages in catalysts is critical. These strong bonds electronically and structurally constrain the La centers, raising the energy barrier for their extraction and chlorination. This confinement maintains the local LaOCl*_x_* coordination environment, preventing the cascade toward LaCl_3_ formation, thereby preserving its ability to stabilize •Cl and activate C_2_H_5_Cl selectively. The catalytic behavior of LaAl_2_O_3_ corroborates these structural findings. Under chlorination conditions of 260 °C and a C_2_H_6_/Cl_2_ molar ratio of 4:9, LaAl_2_O_3_ maintains 61% C_2_H_6_ conversion with a stable 1,2‐C_2_H_4_Cl_2_ selectivity of 74% over 12 h. This stands in contrast to the rapid selectivity decline observed in bulk LaOCl systems.

The ability of rare‐earth oxychlorides to mediate selective chlorination is considered analogous to that observed in CH_4_ chlorination. For LaOCl‐catalyzed oxidative chlorination of CH_4_, a mechanism involving activation of surface chlorine by gas‐phase O_2_ to form OCl^−^ species was proposed, where the formal oxidation state of chlorine, rather than the La^3+^ center, cycles during the reaction [42]. This highlights the role of the oxychloride phase as a stable platform for chlorine redox chemistry. Consequently, the observed transformation of LaOCl to LaCl_3_ under Cl_2_‐rich conditions during C_2_H_6_ chlorination likely represents a shift away from this catalytically active phase, underscoring the critical need for phase stabilization, as achieved in supported systems like LaAl_2_O_3_.

### 3.2 Coupled Gas‐Phase •Cl and Surface‐Catalyzed Reactivity

**FIGURE 6:**
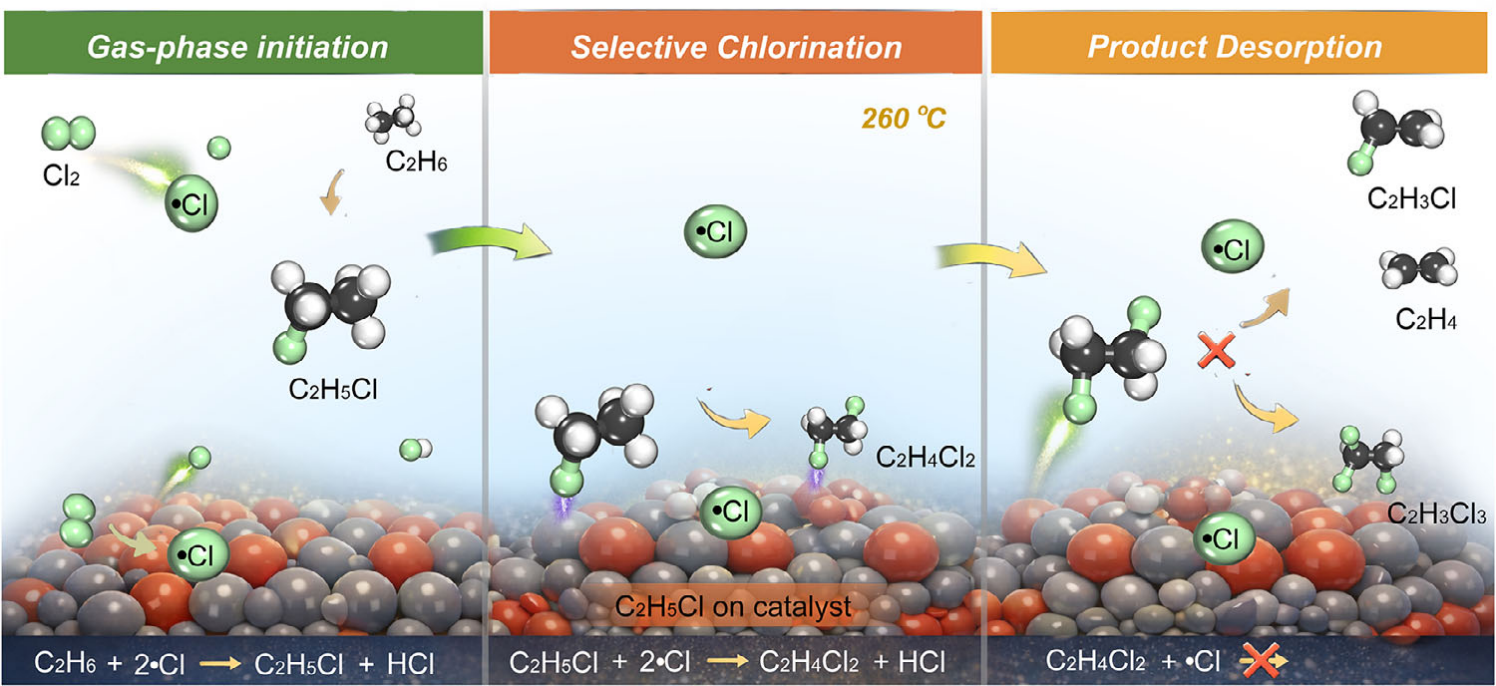
Schematic illustration of the synergistic gas‐phase •Cl and surface‐catalyzed mechanism involved in C_2_H_6_ chlorination.

The experimental and theoretical insights presented in previous sections rationalize the synergistic role of gas‐phase radicals and the catalyst surface (Figure 6). The initiation of the reaction sequence—the activation of the strong C–H bonds in C_2_H_6_— proves challenging for most solid surfaces under mild conditions. In contrast, •Cl, whether generated thermally or via surface‐assisted Cl_2_ activation, abstracts hydrogen from C_2_H_6_ with a very low kinetic barrier. Consequently, the conversion of C_2_H_6_ to C_2_H_5_Cl is predominantly a gas‐phase, •Cl‐mediated process. This explains the frequently observed decoupling between catalyst activity: a catalyst may efficiently generate 1,2‐C_2_H_4_Cl_2_ at lower temperature, yet the conversion of C_2_H_6_ remains limited by the kinetics of radical attack in the gas phase.

The catalyst's pivotal role is unlocked upon the formation of the C_2_H_5_Cl intermediate. The introduction of a polar C–Cl bond enables a strong electronic interaction between C_2_H_5_Cl and the catalyst surface, typically through coordination of the Cl to Lewis‐acidic metal sites (e.g., La^3+^ in LaOCl). This adsorption localizes and activates C_2_H_5_Cl for subsequent transformation. Importantly, the catalyst creates an alternative, lower‐temperature route. By stabilizing reactive chlorine species and adsorbing C_2_H_5_Cl, it enables the second chlorination step to proceed efficiently at a lower temperature (∼260 °C). This critical downshift in the operational temperature window for the selectivity‐determining step is the key to suppressing undesirable gas‐phase pathways. In the absence of such surface mediation, the sequential chlorination of 1,2‐C_2_H_4_Cl_2_ to C_2_H_3_Cl_3_, along with competing dehydrochlorination to C_2_H_2_, are both propagated by •Cl and become significant at elevated temperatures (≥ 300 °C).

## 4 Techno‐Economic and Environmental Evaluation

The transition from traditional C_2_H_4_‐based VCM production to emerging C_2_H_6_‐based routes is increasingly justified by compelling techno‐economic and environmental arguments. While the established business‐as‐usual (BAU) process—based on C_2_H_4_ chlorination and oxychlorination—has historically dominated the global VCM supply, its economic and environmental sustainability is being challenged by the volatility of petroleum markets and the need for carbon footprint mitigation. The evaluation of catalytic C_2_H_6_ chlorination technologies, therefore, involves a dual perspective: quantifying production costs and assessing lifecycle climate impacts under both current and projected energy systems.

**FIGURE 7:**
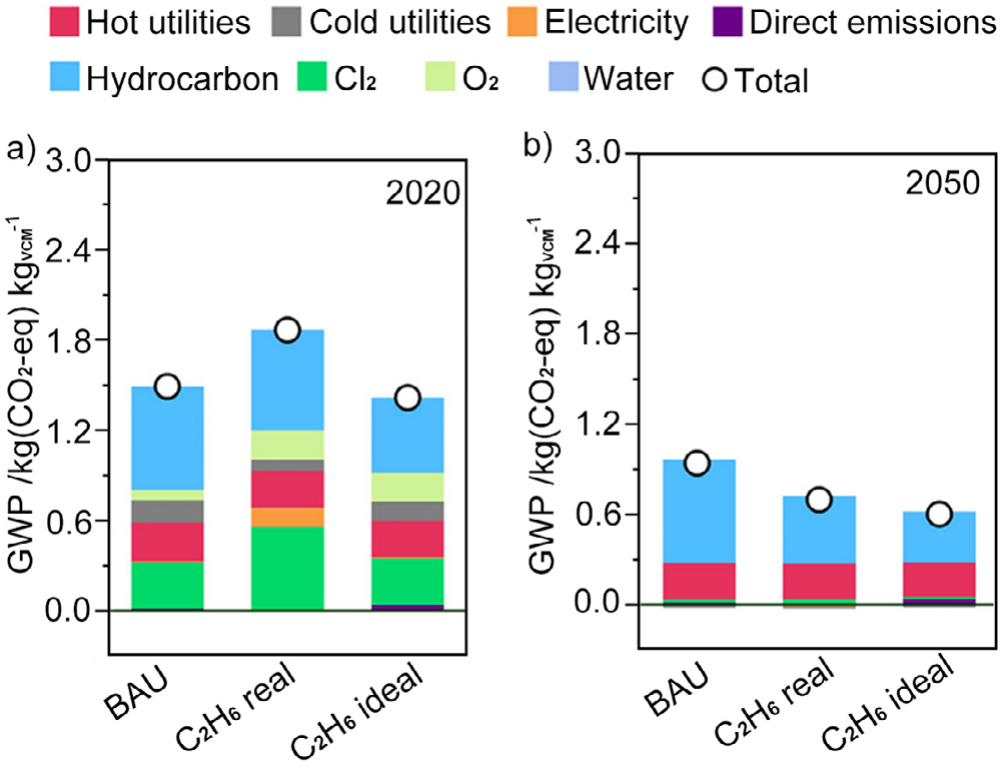
Climate change impact breakdown of the two routes for VCM synthesis (BAU and C_2_H_6_ route, considering both the real and ideal scenarios) for both present (a) and future (b) temporal scenarios. The “hydrocarbon” contribution refers to C_2_H_4_ and C_2_H_6_ for the BAU and C_2_H_6_‐based routes, respectively. Figure adapted from ref [23] with permission.

From an environmental standpoint, the BAU route exhibits a global warming potential (GWP) of 1.87 kg CO_2_‐eq kg^−1^ VCM under the current 2020 energy scenario [23]. This impact arises primarily from C_2_H_4_ feedstock (46%), Cl_2_ (20%), and O_2_ (4%), with heating and cooling utilities accounting for 17% and 10%, respectively (Figure 7a). In comparison, the C_2_H_6_ route operated under realistic conditions—based on experimentally demonstrated catalytic performance (∼90% selectivity at ∼20% C_2_H_6_ conversion)—yields a moderately lower impact of 1.49 kg CO_2_‐eq kg^−1^ VCM, with raw materials (C_2_H_6_, Cl_2_, O_2_) contributing 36%, 30%, and 10%, respectively. Under ideal conditions (assuming 100% conversion and selectivity), the C_2_H_6_ route further reduces the GWP to 1.42 kg CO_2_‐eq kg^−1^ VCM, with raw materials contributing similarly but at lower absolute levels [23].

The environmental advantage of C_2_H_6_‐based routes becomes more evident when prospective energy system decarbonization is considered [23]. Under the 2050 scenario aligned with the Paris Agreement (1.5 °C warming limit), the GWP of the BAU route falls to 0.95 kg CO_2_‐eq kg^−1^, driven by improvements in the electricity mix and increased use of carbon capture technologies (Figure 7b). Still, the C_2_H_6_ route outperforms this benchmark, achieving a GWP of 0.70 kg CO_2_‐eq kg^−1^. Under ideal conditions, its GWP is further reduced to 0.60 kg CO_2_‐eq kg^−1^. This breakdown of environmental impacts highlights the central role of feedstock origin. In the 2050 scenario, C_2_H_4_ accounts for 73% of the BAU's GWP, whereas C_2_H_6_ contributes 72% to the C_2_H_6_ route (Figure 7b) [23].

Utility‐related emissions are sensitive to energy source assumptions. The persistent large contribution of heating, 26% for BAU and 34% for the C_2_H_6_ real route in the 2050 scenario, stems from the continued fossil fuel use. This reveals a significant opportunity for further decarbonization. Electrifying process heating (e.g., cracking furnaces) using the projected carbon‐neutral electricity mix of 2050 could reduce the ‘heating’ portion of the GWP to near zero. A preliminary estimate suggests this alone could lower the total GWP of the C_2_H_6_ real route by approximately an additional one‐third, from 0.70 to about 0.46 kg CO_2_‐eq kg^−1^ VCM [23]. Thus, coupling catalyst innovation with the electrification of thermal units is paramount for achieving deep decarbonization of VCM production.

**FIGURE 8:**
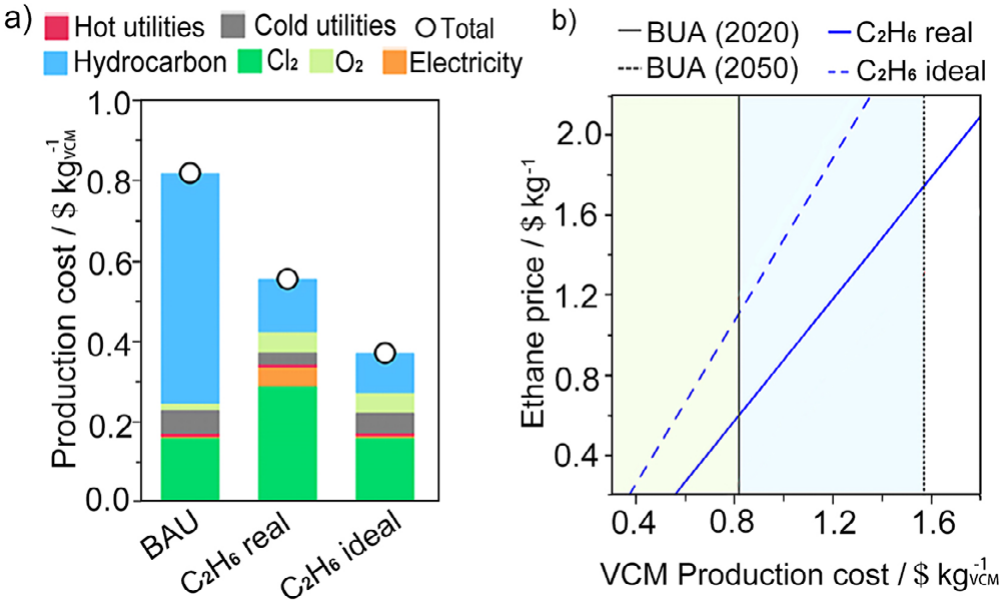
(a) Economic results of the two routes for VCM synthesis (BAU and C_2_H_6_ routes). The “hydrocarbon” contribution refers to C_2_H_4_ and C_2_H_6_ for the BAU and C_2_H_6_‐based route scenarios, respectively. (b) VCM production cost dependence on the C_2_H_6_ price of the two synthesis routes. The economic advantage of C_2_H_6_ chlorination technologies over the BAU is highlighted: green for the current scenario and blue for the prospective scenario. Figure adapted from ref [23] with permission.

Chlorine's marginal impact (2%–4%) reflects its relatively modest energy footprint when derived from modern chloralkali processes powered by renewable electricity. Sensitivity analyses further demonstrate the robustness of the C_2_H_6_ routes to feedstock volatility. While the BAU cost escalates sharply with C_2_H_4_ price (Figure 8a), the C_2_H_6_‐based processes remain economically viable across a broad range of C_2_H_6_ price points. For instance, the C_2_H_6_ route remains cheaper than BAU even with a threefold increase in C_2_H_6_ price (Figure 8b) [23].

Environmental competitiveness, however, depends on achieving sufficiently high product selectivity. At a fixed conversion (∼20%), increasing selectivity to 89% would render the C_2_H_6_ routes environmentally preferable to BAU even in today's energy context [23]. This is because most environmental burdens associated with utilities are driven by separation processes needed to remove byproducts. Therefore, integrated improvements in both catalyst development and reactor design (e.g. Cl_2_ stage‐feeding strategies) are crucial to close the remaining 14%–22% selectivity gap and unlock the full environmental benefits. It is important to note that this selectivity threshold of ∼89% for the C_2_H_6_ route to outperform the BAU environmentally is calculated under the assumption of fixed C_2_H_6_ conversion. In practice, a key catalytic challenge is the trade‐off between conversion and selectivity; achieving higher conversion while maintaining high selectivity is essential for overall process efficiency. Future, more detailed techno‐economic analyses that explicitly couple conversion and selectivity would offer more precise guidance for process optimization.

Importantly, the techno‐economic viability of C_2_H_6_‐based VCM production also depends on the efficiency with which the chlorine loop is closed. Two distinct HCl recycling strategies can be considered: direct C_2_H_6_ oxychlorination and a two‐step process combining Deacon oxidation with separate C_2_H_6_ chlorination. In the direct route, HCl and O_2_ are co‐fed with C_2_H_6_, ideally following reactions such as C_2_H_6_ + 2HCl + ½O_2_ → C_2_H_4_Cl_2_ + H_2_O, but in practice O_2_ simultaneously promotes deep oxidation (C_2_H_6_ + O_2_ → CO*_x_* + H_2_O). Even state‐of‐the‐art oxychlorination catalysts typically achieve < 50% selectivity to VCM [43], translating into higher separation costs and inferior carbon efficiency. Notably, direct C_2_H_6_ oxychlorination generally requires elevated temperatures above ∼450 °C to activate the C_2_H_6_. Under such conditions, the simultaneous presence of HCl, O_2_, and H_2_O creates an exceptionally aggressive environment that accelerates catalyst deactivation. In contrast, the two‐step route decouples oxidation and chlorination. HCl is first oxidized via the Deacon reaction (4HCl + O_2_ → 2Cl_2_ + 2H_2_O), after which C_2_H_6_ chlorination proceeds in an O_2_‐free environment, followed by highly selective 1,2‐C_2_H_4_Cl_2_ cracking to VCM (> 99%). This separation enables selectivity > 80% to 1,2‐C_2_H_4_Cl_2_ and allows independent optimization of each reactor. Although involving more unit operations, the two‐step strategy aligns with established VCM plant architectures and relies on mature technologies, making it the more reliable pathway for closing the chlorine loop.

## 5 Outlook

The catalytic chlorination of C_2_H_6_ has emerged as a compelling alternative to the conventional C_2_H_4_‐based route for VCM production, driven by resource availability and decarbonization imperatives. Despite recent progress, however, the field remains at an early stage of development. Translation from laboratory‐scale demonstrations to industrially viable technologies is hindered by interconnected challenges spanning •Cl‐mediated reaction pathways and catalyst durability. Addressing these challenges requires a shift from empirical optimization toward a more mechanistically grounded research framework.

A key limitation of the current study is the insufficient molecular‐level understanding of •Cl‐mediated chlorination on the catalyst surface. Compared to the extensive study on CH_4_ chlorination, systematic studies of C_2_H_6_ chlorination remain scarce, particularly with respect to the electronic structure and kinetics of surface‐associated •Cl species. Consequently, the activation and transformation pathways of Cl_2_ and chlorinated intermediates on catalyst surfaces remain poorly understood. This gap hampers rational interpretation of experimental selectivity trends, such as the near‐complete suppression of 1,1‐C_2_H_4_Cl_2_ formation on catalyst surfaces despite its thermodynamic accessibility in the gas phase. Bridging this gap will require the combined application of surface‐sensitive characterization and theory‐driven kinetic modeling, with explicit consideration of radical–surface interactions and their temporal evolution under reaction conditions.

From a catalyst design standpoint, current research has focused predominantly on rare‐earth oxychloride systems, which exhibit high selectivity and resistance to deep chlorination. While these materials establish an important performance benchmark, their reliance on rare‐earth elements raises legitimate concerns regarding cost, supply robustness, and long‐term scalability. In this context, insights from earlier studies on CH_4_ chlorination offer valuable catalyst design principles. Foundational studies on solid acids, including zeolites and sulfated metal oxides, showed that electrophilic chlorination pathways can be promoted at the expense of uncontrolled radical chain reactions, enabling selective mono‐chlorination [44, 45, 46]. Translating these design principles to C_2_H_6_ chlorination, while mitigating catalyst sintering and structural degradation, represents a promising route toward expanding the catalyst landscape beyond rare‐earth‐based materials.

Equally critical, yet comparatively underexplored, is the role of reactor engineering in governing selectivity and stability. C_2_H_6_ chlorination is intrinsically dominated by highly exothermic radical reactions, rendering precise control over temperature and reactant concentration profiles indispensable. The widespread reliance on fixed‐bed reactors with uniform feed composition limits the ability to regulate axial Cl_2_/C_2_H_6_ ratios and suppress secondary chlorination. Future progress will likely depend on reactor concepts that enable spatial and temporal control of chlorine availability, such as staged Cl_2_ feeding, dynamic residence time modulation, and the incorporation of quenching or dilution zones.

Given the geographic abundance of natural gas—particularly in North America and the Middle East, which together account for over 48 million tons of C_2_H_6_ annually—C_2_H_6_ offers a regionally secure and economically attractive alternative to oil‐derived C_2_H_4_. On the environmental front, utilizing C_2_H_6_ aligns with long‐term decarbonization strategies, especially when renewable‐sourced Cl_2_ and electrified heating become mainstream. In this sense, the adoption of C_2_H_6_ chlorination technologies supports the dual objectives of reducing reliance on petroleum‐derived feedstocks and meeting carbon mitigation targets without the need for policy enforcement. In conclusion, emerging C_2_H_6_‐based routes for VCM production present a compelling case for both environmental sustainability and economic resilience. Realizing this potential will require sustained, interdisciplinary efforts and a commitment to closing the laboratory‐to‐market gap.

## Conflicts of Interest

The authors declare no conflict of interest.

## Data Availability

The data that support the findings of this study are available from the corresponding author upon reasonable request.
